# Compact folded dipole metasurface for high anomalous reflection angles with low harmonic levels

**DOI:** 10.1038/s41598-020-75230-2

**Published:** 2020-10-22

**Authors:** Nasim Al Islam, Sangjo Choi

**Affiliations:** Department of Electrical Engineering, University of Ulsan, Ulsan, 44610 Korea

**Keywords:** Engineering, Electrical and electronic engineering

## Abstract

A dense unit cell array used in a metasurface for a high reflection angle (θ_r_ > 50°) leads to high coupling among the unit cells; thus, parasitic reflections are unavoidable. The up-do-date patch-based metasurfaces for high reflection angles were electrically large (> 80 λ^2^), but for a practical point of view, a more compact metasurface design is needed. As a solution for these issues, we use the folded dipole-based unit cells with closed-loop currents for low near-field coupling and design compact metasurfaces (~ 40 λ^2^) for high reflection angles (θ_r_ = 56° and 70°) at 10 GHz. The folded dipole unit cells are arranged according to the recently developed non-linear phase boundary condition for low harmonic reflections. As a counterpart, we also designed a metasurface using conventional patch-shaped unit cells with the same reflection phases (θ_r_ = 70°). In experiments, the folded dipole metasurface shows lower harmonic levels (θ_r_ = 0° and − 70°) and a comparable anomalous reflection (θ_r_ = 70°) versus the patch-shaped metasurface. The time-domain analysis demonstrates that the low harmonic levels from the folded dipole metasurface are due to low scattering from the guided waves and the edge scattering. The proposed compact folded dipole-based metasurface with low undesired harmonics can be used as a practical reflect-array for millimeter-wave communication links.

## Introduction

Metasurface is a two-dimensional array of periodic or non-periodic structures that manipulate phase responses at the sub-wavelength scale for wave-front steering in refraction and reflection modes^1–9^. The metasurface concept only requires engineering the structures on a surface instead of a volume and provides certain phase values that determine the wave-fronts of the outcome waves. This straightforward design mechanism of the metasurface has been actively utilized in the development of transmit-arrays^10–12^, reflect-arrays^13,14^, beam-focus lens^15–17^, and vortex beam generators^18,19^.

The relationship between the phase values on the metasurface and the reflection (or refraction) angle was formulated using the generalized Snell’s law^1,20^. This law provides linearly-fashioned phase values on the metasurface with a purely reactive impedance boundary condition but does not guarantee power conservation via impedance matching with the surrounding medium^21^. Here, power conservation means that the net power flow (the normal component of the pointing vector) at any point over the surface is zero; thus, this condition guarantees a perfectly lossless metasurface^21^. However, power conservation for a metasurface with linear phases is only valid in the case of specular reflection in which the angles of incidence and reflection are equal. For the other angles, net power flow becomes nonzero, from which parasitic reflections are created^21^. Furthermore, the undesired reflections become more significant with higher reflection angles (θ_r_ > 50°) under normal incidence (θ_i_ = 0°) and reduce the efficiency of the metasurface, defined by the ratio between the incident power and the reflected power to the desired angle (θ_r_)^21^. One way to reduce harmonics from the metasurface was by adding surface resistance to the boundary, which was derived from an equal amplitude between the incident and reflected waves (E_i_ = E_r_)^21,22^. However, this resistance causes power loss (absorption) on the metasurface, leading towards efficiency drop proportional to a factor of $\cos\uptheta _{{\text{r}}} /\cos\uptheta _{{\text{i}}}$^21^. As the angles of incidence and reflection diverge, the efficiency reduces significantly.

Recently, this issue was resolved by using a boundary condition with the assumption of power conservation (E_i_ $\sqrt {{\cos}\uptheta _{{\text{i}}} }$ = E_r_ $\sqrt {{\cos}\uptheta _{{\text{r}}} }$), where the incident and reflected power are equal relative to their associated angles^21,22^. This boundary condition leads to a complex surface impedance boundary with periodic positive (lossy) and negative (active) resistance, which is equivalent to a non-linear reflection phase distribution as the reflection angle becomes higher^21,22^. In this case, the incident power is fully reflected in the desired angle and ensures almost perfect efficiency even for high reflection angles (θ_r_ > 50°)^21^. Several works adapted the power conservation condition to realize metasurfaces with perfect reflection efficiency through various methods, such as inhomogeneous active and lossy elements^22^, leaky-wave antennas^23^, and coding-based metasurface^24^. Auxiliary evanescent fields in a passive and lossless metasurface also were used to fulfill the power conservation^25^. A metasurface designed by active and lossy elements and auxiliary evanescent field showed highly efficient reflection theoretically, but the physical implementation was not reported. Also, active elements on the metasurface can make structure unstable^22^, and controlling interference of the auxiliary evanescent surface waves between the top and bottom surface of a metasurface makes the design process complex^25^.

Furthermore, the boundary condition for the efficient reflective metasurface was physically implemented by leaky-wave patch-shaped antenna units, which were designed to channel the absorbed power and re-radiate the power inside a period of the cells via surface waves^23^. The designed metasurface (11.7 λ × 7 λ) operating at 8 GHz showed almost perfect reflection from normal incidence to 70°; however, the antenna structure has to be optimized because near-field coupling highly affects the leaky-wave antenna elements^23^. In another work, pre-determined patch-shaped unit cells were arranged in non-linear phase distributions to design efficient reflective metasurfaces (12.8 λ × 10 λ) for high tilt angles (48° and 70°). Measurement results showed efficient reflection at the desired angles, but significant parasitic reflections were shown for the 70° case^24^. From both reports, we can understand that maintaining low parasitic levels in the reflective metasurface for such a high 70° angle is difficult due to the required short distance between the unit cells. The dense unit cell is subject to strong near-field coupling and the phases of the unit cells calculated in the unit cell simulation may not be maintained in the array. Therefore, optimization of the unit cells in the array becomes necessary. Furthermore, a compact metasurface design for practical applications instead of ideal cases such as electrically large metasurfaces (> 80 λ^2^) from both papers would be more challenging because the fringing effect additionally perturbs the near-field of the unit cells and surface waves scatter at the edges. To resolve this issue and avoid optimization burden at the array level, a unit cell structure that can minimize the coupling even in a dense environment can be used. Previously, various shapes such as a V-shape^1^, a H-shape^26^, a circular patch shape^14^, and chiral shapes^27,28^ have been utilized as metasurface unit cells, but these structures may not be adequate inside the dense array due to their bulkier sizes compared to a patch shape^23–25,29^.

In this paper, we utilized a folded dipole structure with the strong self-confined field as a metasurface unit cell and demonstrated efficient reflective metasurfaces (reflection angles > 50°) with low harmonic levels in a compact size (~ 40 λ^2^). The folded dipole structures in the metasurface support closed current loops so that the reflection phases calculated from the unit cell simulation can be maintained even in a dense array. Therefore, the folded dipole-based metasurface did not require any optimization process at the array level. Using the folded dipole unit cells, we designed compact-sized metasurfaces (~ 40 λ^2^) according to the non-linear phase distribution that is required to reflect the normal incident wave towards 56° and 70° with low parasitic reflections. To demonstrate the robust performance of the folded dipole in a dense metasurface, a counterpart structure using conventional patch-shaped unit cells was designed for a 70° reflection angle with the same reflection phases. Then, both metasurfaces for 70° reflection angles were fabricated and measured. The measurement results showed that the folded dipole metasurface maintained lower parasitic reflection levels at (0° and − 70°) and a comparable signal at an anomalous reflection angle of 70° versus the patch-based metasurface. To understand the source of the parasitic signal difference from both metasurfaces, we analyzed the time-domain signals of the reflected waves and found that higher scattering from the guided waves and edge scattering from the patch-based metasurface contribute to the higher parasitic reflection. The folded dipole structure showed a lower number of ringing signals after the main reflection peak, indicating that the lower energy is guided and scattered as undesired harmonics. Due to the robust harmonic suppression performance of the folded dipole-based metasurface in a dense and compact environment, the folded dipole-based metasurface can be utilized as an efficient reflect-array for millimeter-wave communication links.

## Theory and metasurface design

### Theoretical analysis

A metasurface design method from the generalized Snell’s law provides a phase gradient (slope) on a surface boundary for a specific wave bending angle in both the reflection and refraction modes^1^. The phase gradient along 2π phases determines a period (D_x_) of the metasurface and formula of D_x_ for reflecting a plane wave with an incident angle of $\uptheta _{{\text{i}}}$ to a reflection angle of $\uptheta _{{\text{r}}}$, is given by

1 $${\text{D}}_{{\text{x}}} = \frac{\uplambda }{{\left| {{\sin}\uptheta _{{\text{r}}} - {\sin}\uptheta _{{\text{i}}} } \right|}}$$

where λ is the operating wavelength. Here, the phase variation over one period of the metasurface along the x-axis ($\upphi \left( x \right) = 2\uppi {\text{x}}/{\text{D}}_{{\text{x}}}$) is the tangential wavenumber difference at the boundary multiplied by the x position (k(sinθ_r_ − sinθ_i_)x). If the reflection coefficient is assumed to have a unity magnitude, the surface impedance (Z_s1_), defined by the ratio between the tangential component of the total electric and magnetic field at the surface can be represented by

2 $${\text{Z}}_{{{\text{s}}1}} = {\text{j}}\frac{\upeta }{{\cos\uptheta _{{\text{i}}} }}\cot \left[ {\upphi \left( {\text{x}} \right)/2} \right]$$

where $\upeta$ is the intrinsic impedance of free space as a medium above the metasurface. Because this surface impedance was not derived from Maxwell’s equations, the condition of the unity magnitude (|E_i_| =|E_r_|) cannot be satisfied without allowing undesired reflected waves to the other angles; thus power conservation cannot be maintained^25^. Instead, the surface impedance can be calculated using Maxwell’s equations and the power conservation condition (|E_i_|$\sqrt {cos\theta_{i} }$ =|E_r_|$\sqrt {cos\theta_{r} }$) where the power carried out by the incident wave and the reflected wave at the desired angle are equal^25^. When the incident wave is in TE mode, the required surface impedance (Z_s2_) can be derived as

3 $${\text{Z}}_{{{\text{s}}2}} = \frac{\upeta }{{\sqrt {\cos\uptheta _{{\text{i}}} \cos\uptheta _{{\text{r}}} } }}\frac{{\sqrt {\cos\uptheta _{{\text{r}}} } + \sqrt {\cos\uptheta _{{\text{i}}} } {\text{e}}^{{{\text{j}}\upphi \left( {\text{x}} \right)}} }}{{\sqrt {\cos\uptheta _{{\text{i}}} } - \sqrt {\cos\uptheta _{{\text{r}}} } {\text{e}}^{{{\text{j}}\upphi \left( {\text{x}} \right)}} }}$$

Different from the only reactive surface impedance from Z_s1_, Z_s2_ becomes complex with periodic surface resistance with positive (lossy) and negative (active) values along the x-axis. Finally, the Z_s1, 2_ values can be converted to reflection coefficients (R_1,2_) according to

4 $${\text{R}}_{1,2} = \frac{{{\text{Z}}_{{{\text{s}}1,2}} -\upeta }}{{{\text{Z}}_{{{\text{s}}1,2}} +\upeta }}$$

**Figure 1:**
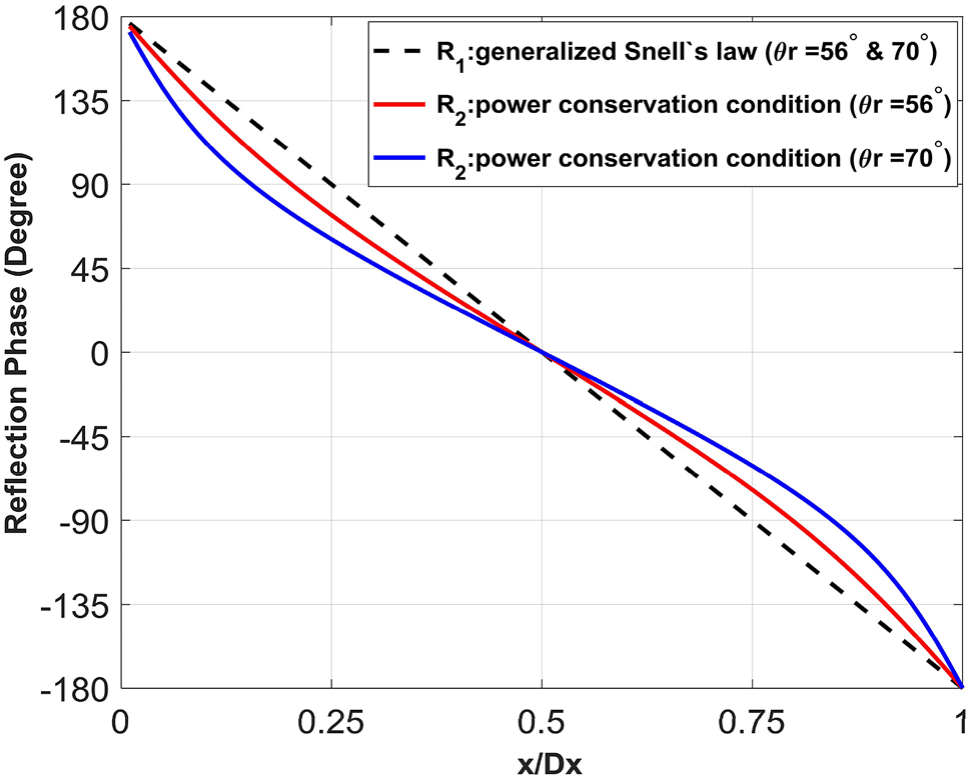
Reflection phase distribution for one period of metasurfaces to reflect a normal incident plane wave into reflection angles of 56° and 70° (black dashed line: R_1_ from the generalized Snell’s law, blue and red solid lines: R_2_ from the power conservation condition).

We chose two high reflection angles (θ_r_ = 56° and 70°) with normal incidence (θ_i_ = 0°) and calculated the surface impedance values (Z_s1,2_) according to Eqs. (2) and (3). Then, the reflection phases (R_1,2_) over one period (D_x_) of the surface boundaries were found according to Eq. (4) and plotted as a function of the normalized period (x/D_x_) in Fig. 1. The R_1_ values for both reflection angles are linear with a constant slope, while the R_2_ values are non-linear and the nonlinearity becomes higher as the reflection angle increases.

### Folded dipole unit cell design

**Figure 2:**
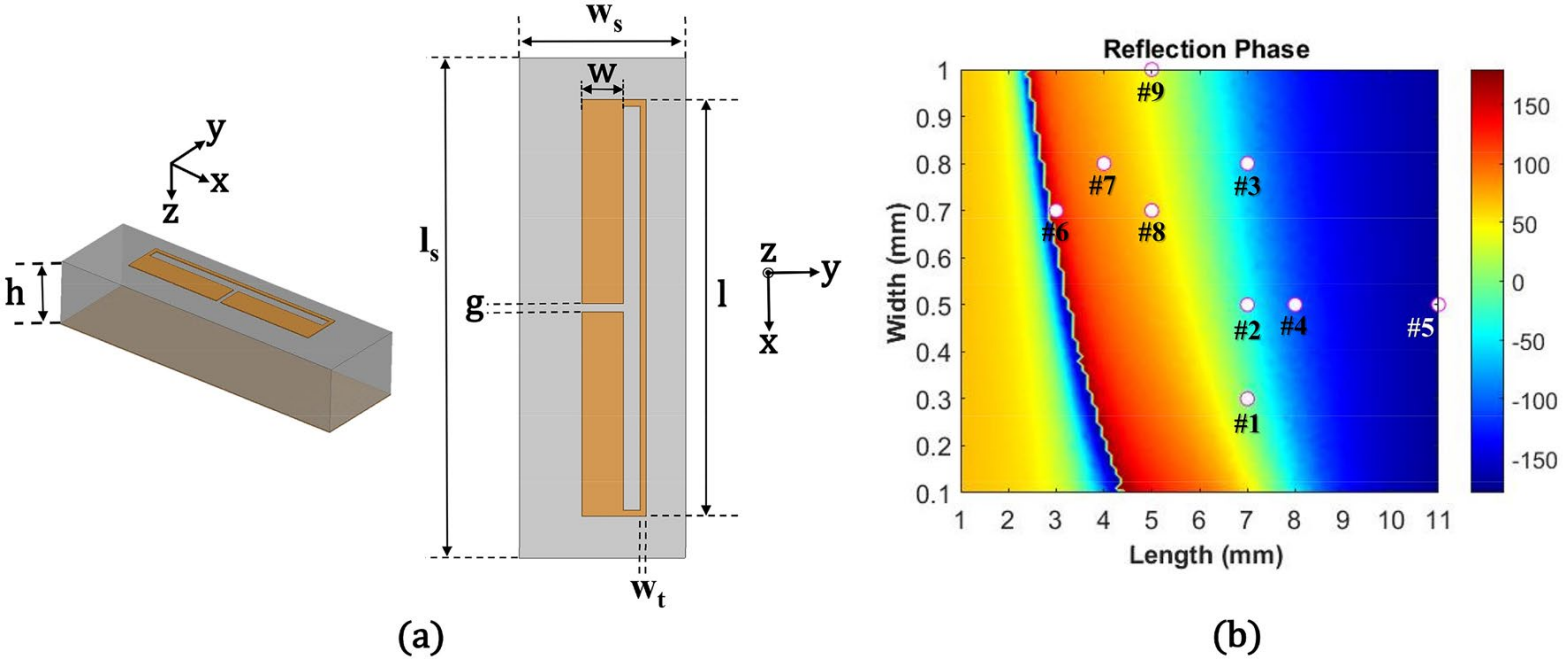
(**a**) Schematic of a folded dipole unit cell with geometrical dimensions. (**b**) Reflection phases in degrees at 10 GHz as a function of length (l) and width (w) of the folded dipole structure for 70° reflective metasurface (w_s_ = 3.5 mm) and the positions of the selected unit cells.

Based on the reflection phases for a specific angle from the power conservation condition, we designed reflective metasurfaces using folded dipole shaped structures operating at 10 GHz. Figure 2a shows a schematic of a folded dipole unit cell and geometrical dimensions based on Rogers 6006 with 2.5 mm thickness (h). Here, a metallic ground plane under the substrate was utilized in the unit cell design. By using Eq. (1), we found that required period lengths (D_x_) were 36 mm and 32 mm at 10 GHz for the 56° and 70° reflective metasurfaces along the y-direction. With 9 unit cells in one period, these two periods led to the widths (w_s_) of each unit cell of 4 mm and 3.5 mm for 56° and 70° cases, respectively. The length (l_s_) of the unit cell for both metasurfaces was fixed with 12 mm for 2π reflection phase coverage. Therefore, the final dimensions (l_s_ × w_s_ × h) of one unit cell were set to 12 × 4 × 2.5 mm^3^ and 12 × 3.5 × 2.5 mm^3^ for 56° and 70° reflection angles, respectively. Based on the unit cell sizes, we varied the width (w) and length (l) of the folded dipole arms to achieve almost 2π reflection phases at 10 GHz. Here, the width (w_t_) of the connecting trace and the gap size (g) between the metallic arms were fixed with 0.15 mm and 0.2 mm. Two metallic arms with widths of w and w_t_ were connected with a 0.4 mm-long line. With these conditions, the folded dipole structures covered the required phase variation with almost a unity reflection magnitude for both 56° and 70° cases. Figure 2b shows the 2π phase coverage of unit cells at 10 GHz for 70° reflection angle (w_s_ = 3.5 mm) with variations of the length (l) and width (w). Finally, we selected the unit cells according to the phases of R_2_ by changing the length (l) in whole number increments and fine-tuning the width (w) of the folded dipole structures and summarized the dimensions of the selected unit cells in Table. 1. In Fig. 2b, the dimensions of the final unit cells for the 70° reflective metasurface are marked by white dots.

**Table 1:** Dimensions of the unit cells.

Unit cell	56° reflective structure			70° reflective structure		
	l (mm)	w (mm)	Phase (°)	l (mm)	w (mm)	Phase (°)
1	6	0.9	− 1	7	0.3	− 1
2	7	0.6	− 37	7	0.5	− 33
3	8	0.3	− 77	7	0.8	− 68
4	8	1	− 120	8	0.5	− 103
5	11	0.5	− 170	11	0.5	− 170
6	3	0.7	142	3	0.7	146
7	5	0.2	108	4	0.8	78
8	5	0.5	74	5	0.7	55
9	6	0.5	38	5	1	35

### Design and simulation of the folded dipole-based metasurfaces

**Figure 3:**
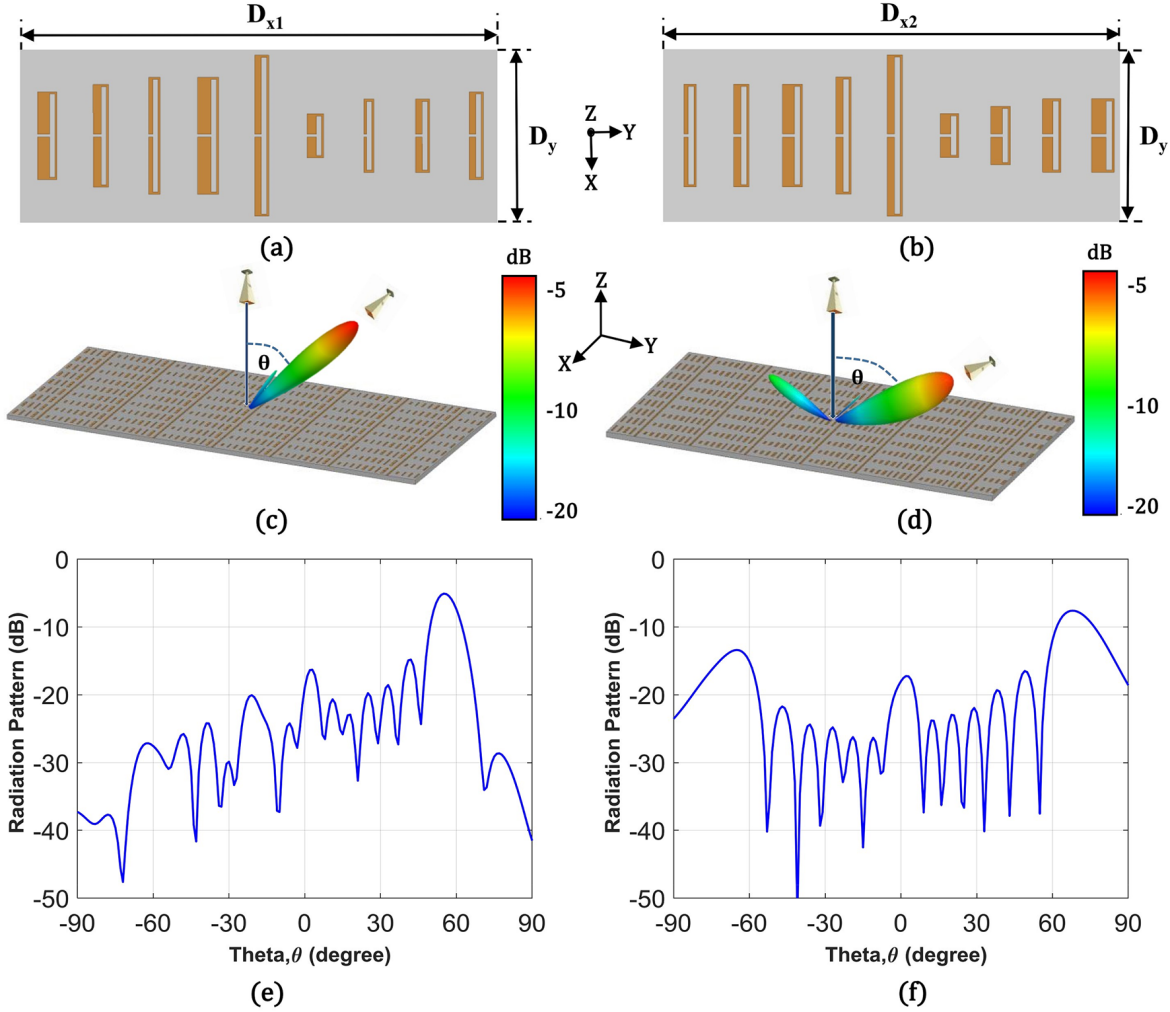
Folded dipole unit cells in one period for (**a**) $\uptheta _{{\text{r}}} =$ 56° and (**b**) $\uptheta _{{\text{r}}} =$ 70°. Far-field 3D radiation pattern at 10 GHz for the finite 7 by 9 periods of (**c**) 56° and (**d**) 70° reflective metasurfaces. Far-field 2D radiation pattern at 10 GHz on $\upphi$ = 90° plane for the finite 7 by 9 periods of (**e**) 56° and (**f**) 70° reflective metasurfaces.

We arranged the unit cells in Table. 1 and realized the periods for two reflective metasurfaces with θ_r_ = 56° and 70°, as shown in Fig. 3a,b. Here, D_x1_ (36 mm) and D_x2_ (32 mm) are the D_x_ values for the 56° and 70° cases, and D_y_ is fixed at l_s_ (12 mm). In full-wave simulations, we excited finite arrays of the metasurfaces with a normally incident plane wave and found the minimum array size that provides reflection beams at desired angles. 7 by 9 periods along the x and y-axis for both cases were chosen, therefore; the finite metasurface sizes became 252 mm × 108 mm (8.4 λ × 3.6 λ) and 220.5 mm × 108 mm (7.35 λ × 3.6 λ) for the 56° and 70° reflection angles. We did not further tune the unit cells in the finite array because we expected that the closed-loop current inside the folded dipole structure to ensure stable phase performance in a dense array. Figure 3c,d demonstrates the simulated 3D radiation patterns from the metasurfaces for θ_r_ = 56° and 70°, indicating clear anomalous reflection beams for both cases. However, the 3D pattern for θ_r_ = 70° case shows a reflected beam to an undesired direction at θ = − 70°. For more detail, the 2D radiation patterns at a plane with $\upphi$ = 90° are plotted in Fig. 3e,f. Figure 3e for the θ_r_ = 56° case shows a negligible reflection level at θ = 0° that is 10 dB lower than the level at θ = 56° and Fig. 3f for θ_r_ = 70° case shows more significant signal levels at θ = 0° and θ = − 70°. Both angles can be understood as undesired harmonic reflections of n = 0 and n = − 1 orders which correspond to 0 or − 2π/D_x_ of the tangential wavenumbers of the reflected wave (nk_0_sinθ_r_). These harmonic levels from the metasurface for θ_r_ = 70° occur because negative resistance required for efficient reflection to high angles was not implemented through the passive folded dipole elements even though the required reflection phases were realized. However, in Fig. 3f, harmonic levels with n = 0 and n = − 1 are still lower than the main peak by 8 dB and 5.3 dB, respectively, which means both power levels are below 30% of the radiated power in the anomalous direction. These harmonic levels are lower than those from a reported patch-based metasurface for 70° reflection angle where the level at θ = − 70° reached 60% of the anomalous reflection level^24^.

### Performance comparison with patch-based metasurface

**Figure 4:**
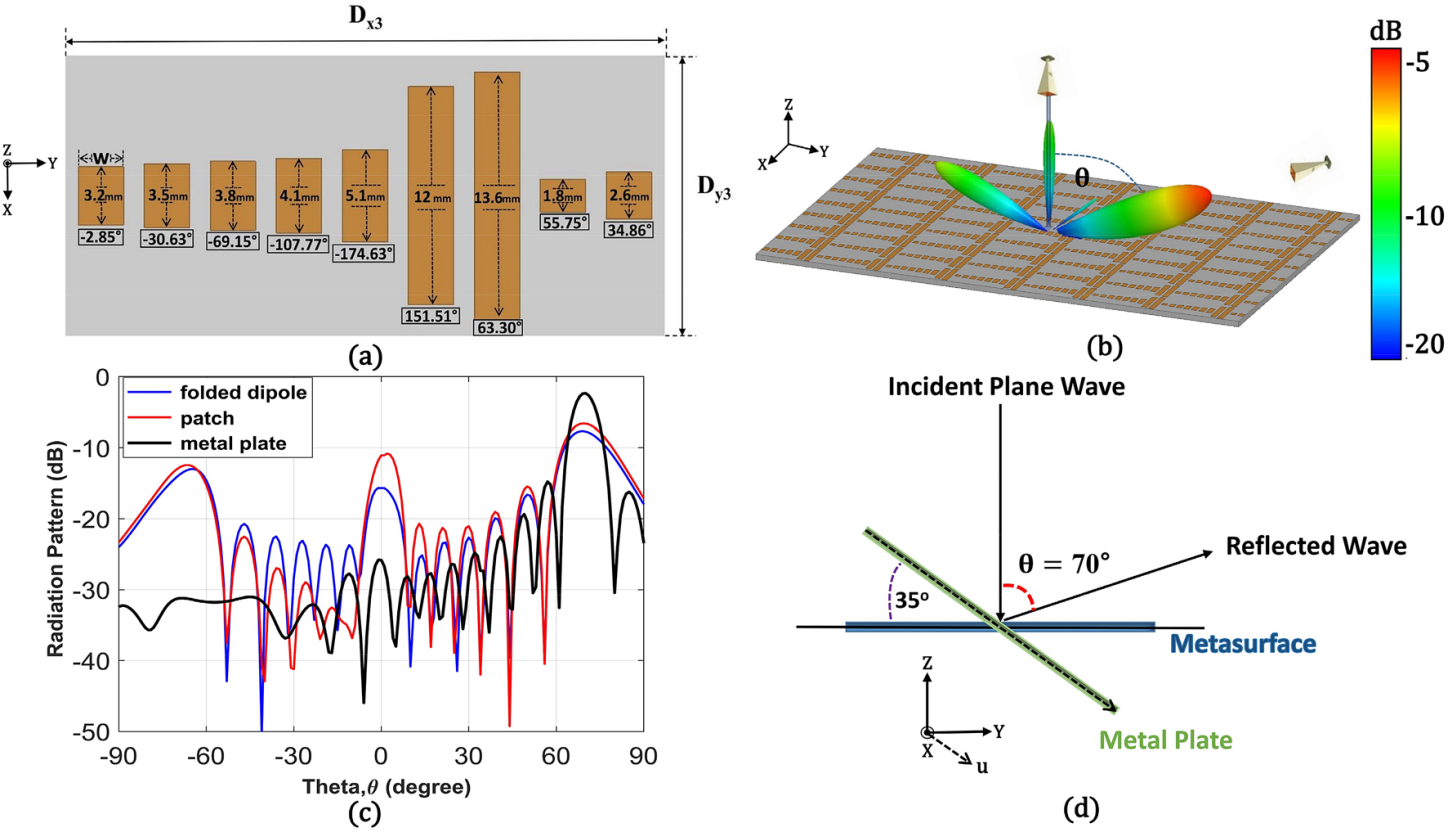
(**a**) Geometry of a patch-shaped 70° reflection metasurface with unit cells’ reflection phases. (**b**) The far-field 3D radiation pattern for a finite 7 by 8 array of 70° reflective patch-shaped structure. (**c**) Comparison of far-field 2D radiation patterns at 10 GHz for the folded dipole, the patch-shaped metasurfaces, and the metal plate (at $\upphi$ = 90° plane). (**d**) The geometry of the simulation setup for measuring specular reflection from a metallic plate compared to a metasurface.

As a counterpart of the folded dipole metasurface, we designed a conventional patch-shaped metasurface for θ_r_ = 70° with the same phase distribution (R_2_) and compared the reflection levels at θ =  + 70°, 0°, and − 70°. In the same unit cell size (12 mm × 3.5 mm), the length of the patch was varied from 1 to 11 mm with a constant patch width (W) of 2.4 mm. Differently from the folded dipole unit cells, the patch-shaped structures covered reflection phases of only 246° (− 180° to + 66°) from the 12 mm-long unit cell. Therefore, we increased the length of the unit cell up to 15 mm to lower the resonance frequency and achieved almost 2π phase variation at 10 GHz. The increased D_y_ by 3 mm led to a 27 mm (0.9 λ) increase for the patch-based metasurface with 7 by 9 periods compared to the folded dipole metasurface. Figure 4a shows the dimensions of the selected unit cells inside one period (D_x3_ = 32 mm and D_y3_ = 15 mm) of the patch-shaped metasurface for 70° reflection angle. For a fair comparison with the folded dipole-based metasurface, we arranged 7 by 8 periods (along the x and y-axis) of the patch-shaped unit cells, and the final metasurface for the 70° bending angle became 220.5 mm × 120 mm (7.35 λ × 4 λ), which is slightly larger than 220.5 mm × 108 mm (7.35 λ × 3.6 λ) of the folded dipole metasurface. Figure 4b demonstrates the 3D radiation pattern from the finite structure simulation and clearly shows anomalous reflection at 70°. However, higher reflected beams at 0° and − 70° compared to the folded dipole-based metasurface can be seen.

Figure 4c compares simulated 2D radiation patterns for both metasurfaces on a plane with $\upphi$ = 90°. Here, a simulation result from a metallic plate is added as a reference for the anomalous reflection levels for both metasurfaces. Figure 4d shows a simulation setup where a metallic plate of the same size as the metasurface is rotated 35° along θ direction. The metallic plate was illuminated with an incident beam at θ = 0° and the specular reflection was used to achieve a reflected beam at θ = 70°. Figure 4c shows the highest reflection peaks at 70° from all the structures and the patch-shaped metasurface showed slightly higher reflection about 1 dB from the folded dipole one. However, the patch-shaped metasurface exhibits higher parasitic reflection at − 70° (n = − 1) by 1.13 dB and at 0° (n = 0) by 5 dB in comparison with the levels from the folded dipole metasurface. The lower harmonic levels from the folded dipole metasurface can be understood as that the desired reflection phases for the 70° anomalous reflection angle were not perturbed much in its dense array. The finite-sized metasurface is also subject to the edge scattering through the guided waves inside the structure. Therefore, different waveguiding mechanisms related to the near-field coupling inside both metasurfaces will also affect the harmonic levels. In the next chapter, related simulation results on the field and current distribution from the metasurfaces will be followed.

### Power flow and surface current analysis

**Figure 5:**
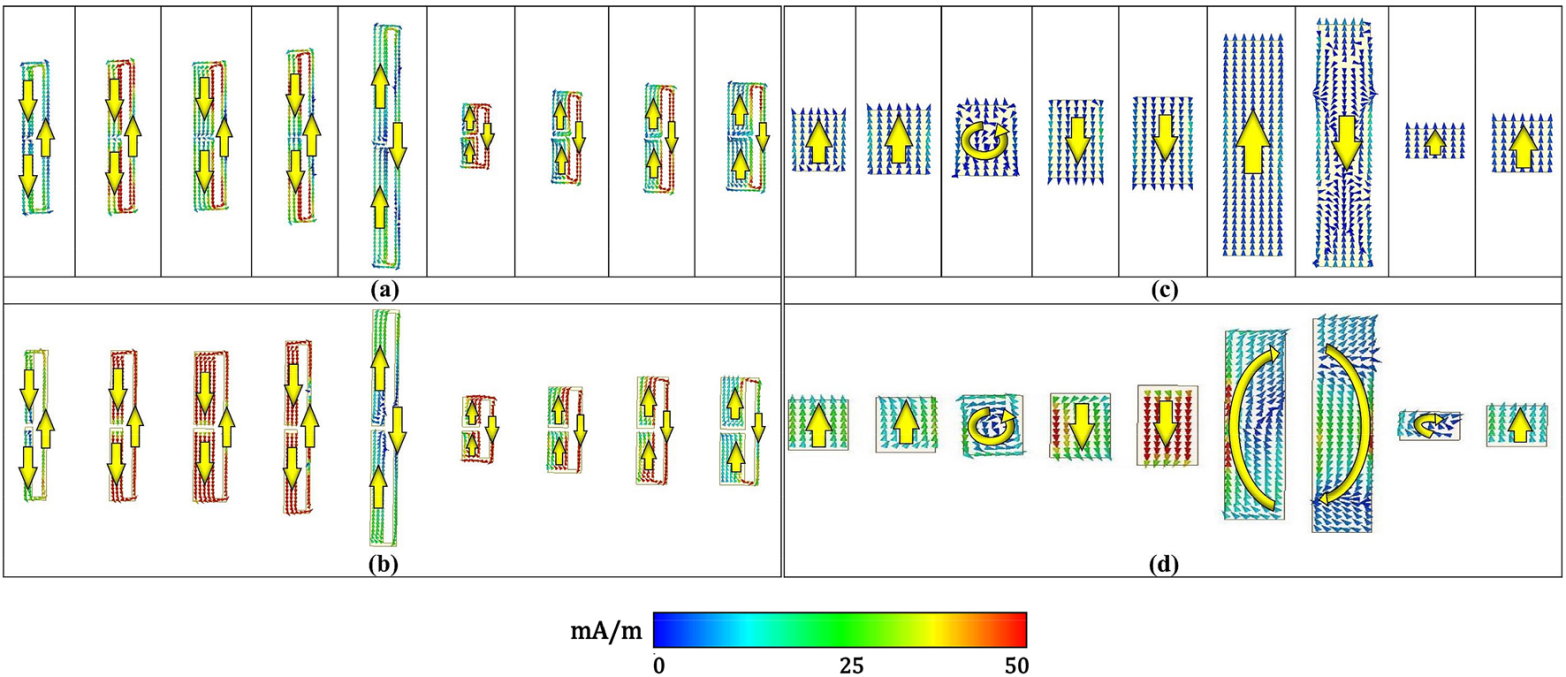
Surface current distribution inside the folded dipole unit cells (**a**, **b**) and the patch-shaped unit cells (**c**, **d**) in the metasurfaces for a 70° reflection angle. (**a**, **c**) are from individual unit cell simulations. (**b**, **d**) are from one period in the finite array simulations.

In this section, we compare the surface current distribution and power flow between the folded dipole and the patch-shaped metasurfaces designed for 70° reflection. Figure 5a shows the surface current flow in unit cell simulations of the folded dipole structures using the periodic boundary condition. We also simulated the surface current distribution on the finite metasurface and presented the results from one period in Fig. 5b. Both cases used the same incident wave setup using plane wave excitation (E = 1 V/m) for a fair comparison. Figure 5a,b clearly shows that closed current loops are excited inside the arms of the folded dipole unit cells, and this trend is also maintained with a minor magnitude difference in a finite array simulation. This result indicates that near field coupling between adjacent cells is not strong and the phase determined from unit cell simulations can be maintained inside the array. Figure 5c,d shows the surface current distribution of the patch-shaped structure with the same simulation setup. Differently from the folded dipole unit cells, the 6th and 7th elements from the finite array simulation mutually generate a circulating current, and 3rd and 8th elements clearly show different current distributions between both cases. The perturbed current distributions from the patch-based metasurface indicate the existence of the strong near field coupling in the array. Therefore, we can understand that the reflection phases inside the patch-based metasurface must be perturbed due to the near-field coupling and this effect may lead to the higher undesired harmonic levels from the patch-based metasurface.

**Figure 6:**
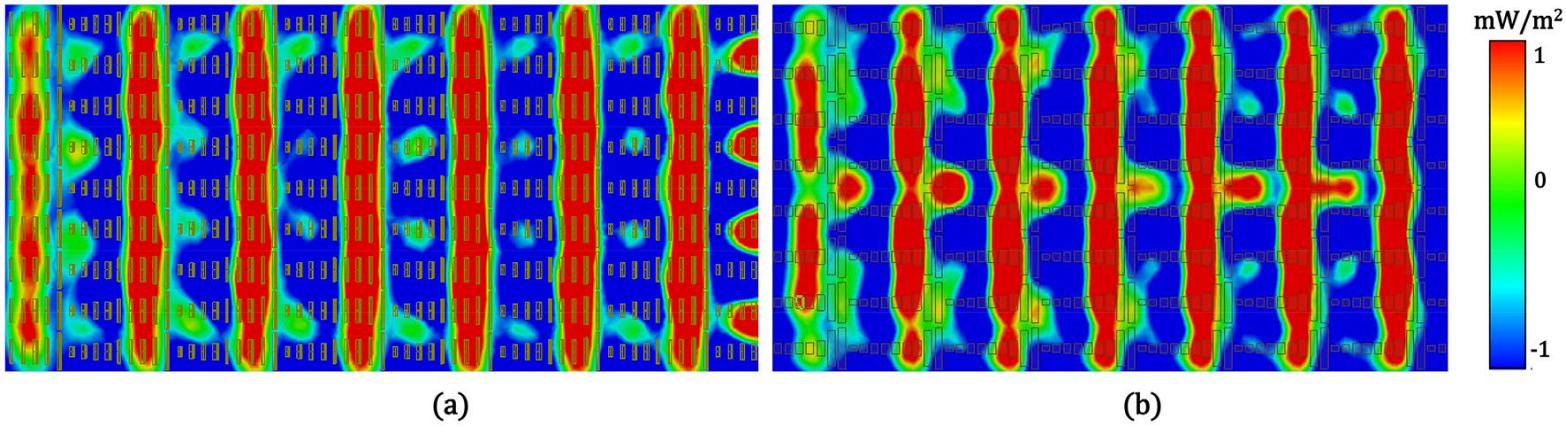
Power flow (Re{S_z_}) on a plane located at z = 4.5 mm (λ/6.67) from (**a**) the folded dipole metasurface and (**b**) the patch-shaped metasurface.

To assess the effect of the perturbed current distribution inside the patch-based metasurface on the reflected power flow, we simulated the z-directed Poynting vector (Re{S_z_}) above the metasurface^30^. Here, the Poynting vector represents the net power flow over the metasurface and indicates where the incident power is radiated or absorbed. Figure 6a,b shows the reflected power flow above the folded dipole and patch-shaped metasurfaces on a plane located at z = 4.5 mm (λ/6.67). Figure 6a shows the periodic distribution of the power density on the surface, where half of the surface is active (Re{S_z_} > 0) and the other half is lossy (Re{S_z_} < 0), ensuring the requirements for efficient anomalous reflection. However, Fig. 6b from the patch-shaped case shows that the radiated power near the center in the active region leaks to the adjacent lossy part, indicating that the near field coupling between the relevant unit cells perturbs the power distribution. This perturbed reflected power flow reinforces the idea that near-field coupling inside the patch-based metasurface leads to higher undesired harmonic levels.

## Experiments and measurement result

**Figure 7:**
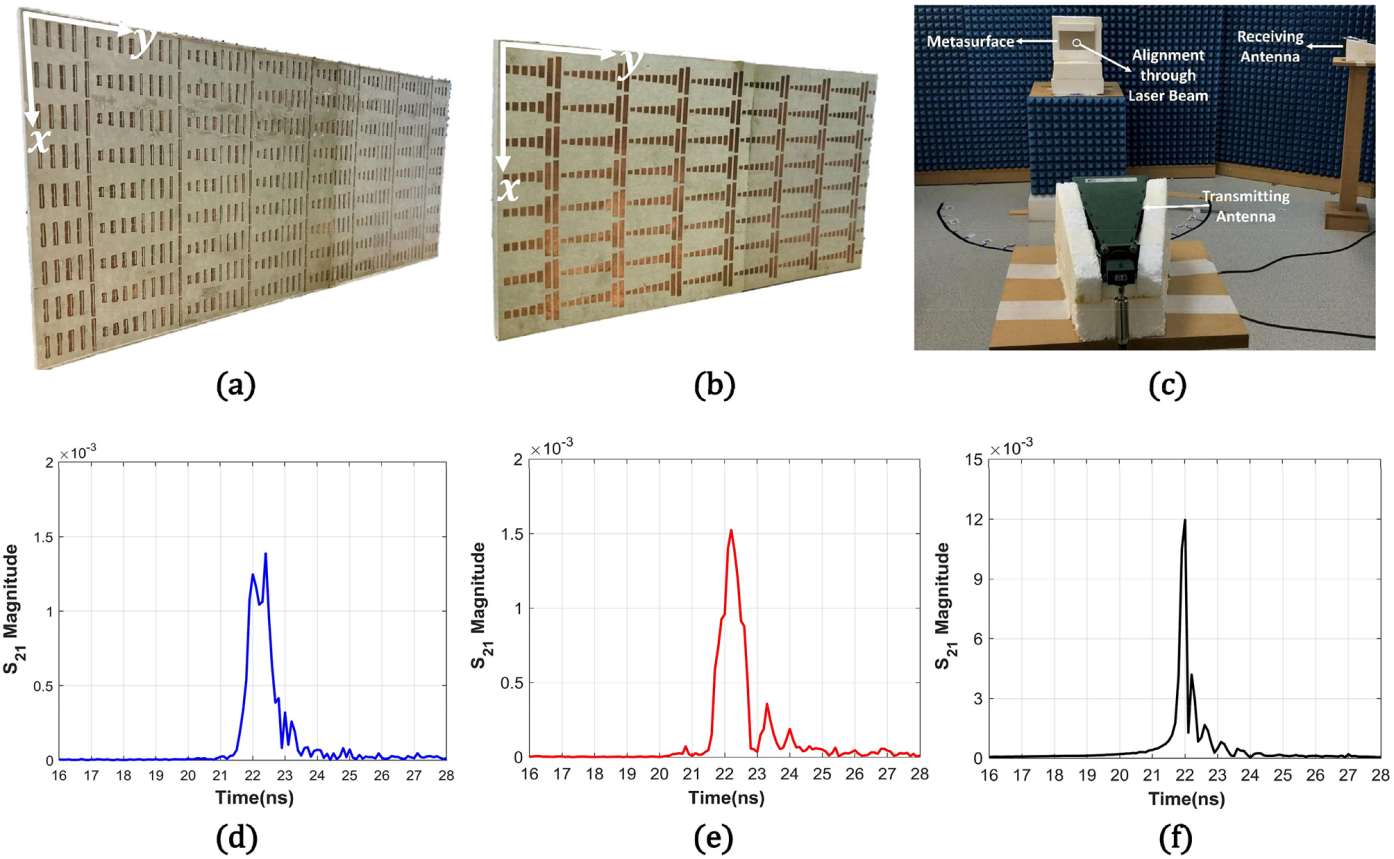
(**a**) Fabricated folded dipole metasurface with 7 by 9 periods. (**b**) Fabricated patch-type metasurface with 7 by 8 periods. (**c**) Measurement setup for the metasurfaces. Time-domain signal of S_21_ measurement results at 70° of (**d**) the folded dipole metasurface and (**e**) the patch-shaped metasurface. (**f**) Time-domain reflected signal at 70° from the metallic plate with a size of the patch-based metasurface when the plate was rotated by 35° relative to θ = 0°.

We fabricated the folded dipole (7 × 9 array) and patch-shaped (7 × 8 array) metasurfaces in 224 mm (width) × 108 mm (length) and 224 mm × 127 mm of standard-sized Rogers 6006 substrates with a 2.5 mm thickness and 35 μm-thick copper, as shown in Fig. 7a,b. Both metasurfaces utilized the same width along the y-axis and slightly different lengths along the x-axis due to different optimum unit cell lengths. A measurement setup in Fig. 7c was used for verification of the reflection performance at three angles (θ = − 70°, 0°, and 70°). In this setup, a transmitting horn antenna was fixed at a 0° angle in front of the metasurface with 4 m distance to ensure that the uniform plane wave impinges on the metasurface. A receiving antenna was mounted at 2 m from the metasurface at two target angles (− 70° and 70°). We confirmed the alignment and angles between the metasurface and the horn antennas using an industrial alignment laser (Easy-Laser 22152). Both horn antennas were connected to a network analyzer (Anritsu MS46122B) and two-port s-parameters were used to measure the reflected signals. For the θ = 0° case, return loss at the port connected to the transmitting antenna was used. Then, wide-band frequency domain data (5–15 GHz) from the network analyzer were collected with 1601 sampling points. We converted the frequency domain signal to the time-domain signal using IFFT (Inverse Fast Fourier Transform).

Figure 7d,e shows the time-domain signal in nanoseconds (ns) for the measured S_21_ at θ = 70° from the folded dipole and patch-based metasurfaces. As references, reflected signals were measured at the same receiving antenna from two metallic plates with the same sizes of two metasurfaces, which were rotated by 35° relative to θ = 0° and data from a metallic plate for the patch-based metasurface is depicted in Fig. 7f. From these figures, we observe that the folded dipole structure shows a reflected signal peak at the same time with the metal plate at 22 ns, indicating direct reflection without any time delay. Because the folded dipole metasurface utilizes the field excited inside the antenna structure (on the surface), the direct reflection becomes possible. However, the patch-shaped structure showed a main peak at 22.2 ns, which exhibits 0.2 ns delay compared to the direct reflection. This means that the maximum reflection from the patch-shaped metasurface is from a guided wave inside a period, not from direct reflection like the other two structures. Though the folded dipole metasurface also showed another peak after 0.2 ns due to the guided wave, it contained weaker subsequent reflection peaks after the main peaks compared to the patch-based metasurface. This phenomenon indicates that guided waves inside the patch-based metasurface scattered at the edges of the structure more significantly.

**Figure 8:**
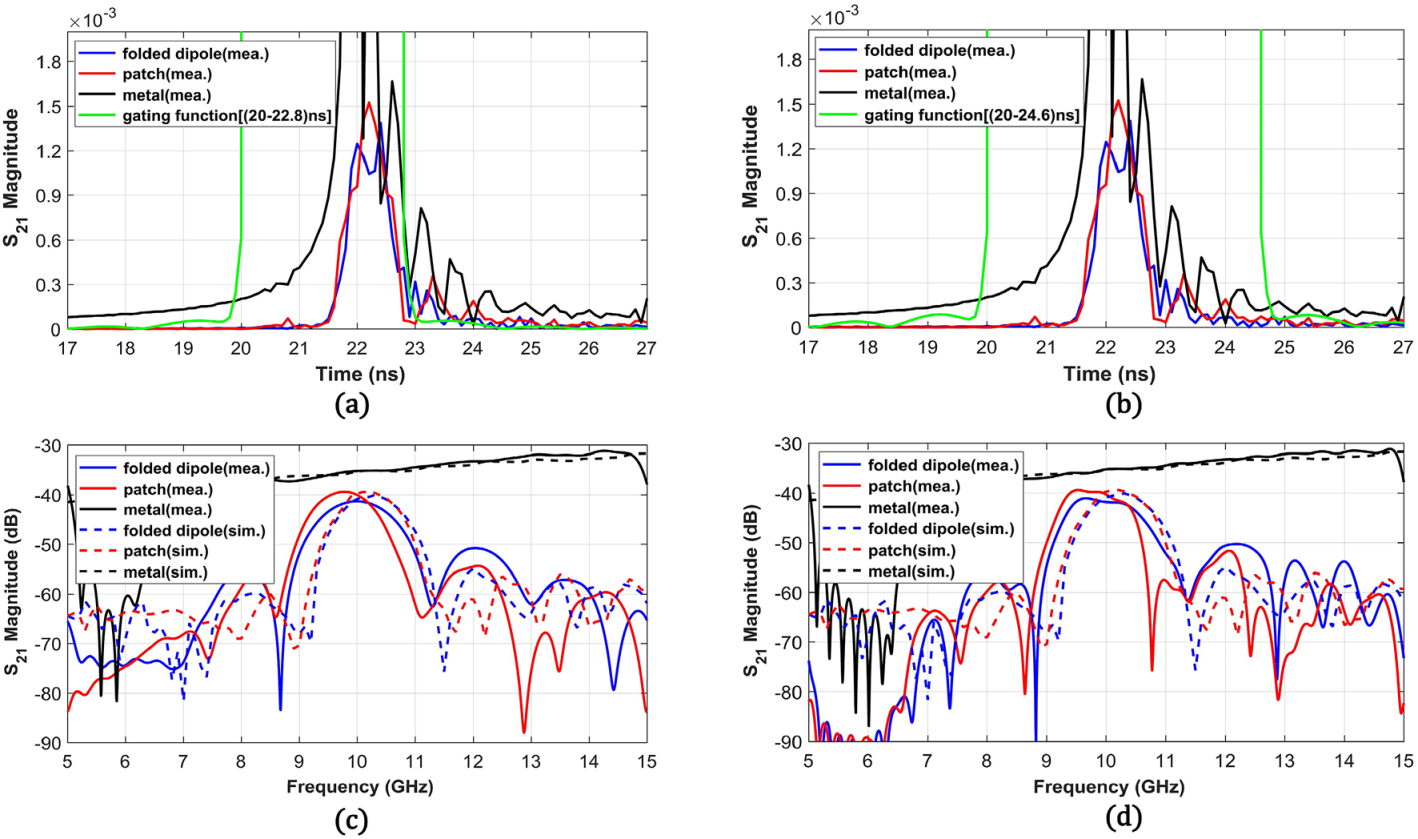
Transmission coefficient (S_21_) at θ = 70° in (**a**) the time-domain with a 2.8 ns-wide gating window (20–22.8 ns) for considering the main peak of reflection from the structures and (**b**) the time-domain signal with a 4.6 ns-wide gating window (20–24.6 ns) for considering the main peak along with the following reflection peaks. Simulation results without the gating and measurement results in the frequency domain converted from the gated time-domain signals using (**c**) the 2.8 ns and (**d**) the 4.6 ns gating windows.

To understand the effect of the scattered wave from finite-sized metasurfaces on the reflection level in the frequency domain, we used the time-gating process through which we can selectively filter desired reflection peaks in the time-domain. We gated the time-domain signal using the Kaiser window-based gating functions with two different lengths (2.8 ns and 4.6 ns) to distinguish the effect from subsequent reflection peaks due to scattering at the edges. The gated signals in the time-domain were then converted to the frequency domain signals by using FFT (Fast Fourier Transform). Figure 8a,b shows the time-domain signals of S_21_ measured at θ = 70° (n = 1) for all three structures and includes the 2.8 ns and 4.6 ns-wide gating functions. The 2.8 ns (20–22.8 ns) gating span only selects the main reflection peak and the 4.6 ns (20–24.6 ns) gating span additionally includes the following peaks. Figure 8c shows the frequency domain signals from the gated signals with the 2.8 ns span, demonstrating a good agreement with the simulation results with a frequency downshift. We expect that the dielectric constant variation in PCB and the higher parasitic effect in the measurement shifted the resonance. Here, the simulation results indicate the radiation pattern at θ = 70° along the frequency domain from the metasurfaces and the maximum peak level is adjusted to the maximum of the measurement result. Figure 8d denotes frequency domain responses from the 4.6 ns-wide gating function and indicates that inclusion of the following reflection peaks in the time-domain led to a signal drop with a steep skirt at an upper band (10.5–11 GHz) from the patch-shaped metasurface. However, the folded dipole metasurface only experienced ripples near in-band. This result demonstrates that the stronger ringing effect inside the patch-shaped metasurface resulted in greater deviation from the simulation results, but the folded dipole-based metasurface maintains a more stable reflection performance in a dense array. Here, we experimentally found that the strong coupling between unit cells and the related ringing effect in a finite metasurface for a high reflection angle can cause signal drops and ripples in the desired reflected beam. Because this complicated behavior is not encountered in the metasurface design using the periodic boundary condition, it is important to note that the unit cell structure with lower coupling can ease the metasurface design, especially in a compact platform for practical applications.

We also investigated the efficiencies of both metasurfaces compared to the reflected power of the metallic plates with the same sizes of metasurfaces at 10 GHz. Note that for the efficiency calculation, we chose the reflection levels from the wider gating span (4.6 ns) which consider more realistic situations. In Fig. 8d, the folded dipole and the patch shaped metasurfaces show reflection levels of − 41.80 dB and − 40.26 dB at 10 GHz with a slight difference with 1.54 dB that corresponds to 1.14 dB difference from simulations. The similar signal difference is also maintained up to near 9.6 GHz. Using the signal levels of metallic plates with the same sizes of the folded dipole and patch-shaped structures as references, we calculated the efficiencies of both metasurfaces using a method described in the Methods section. For the efficiency calculation, a correction factor to compensate for the lower aperture gain of the metasurface compared to the gain from the metallic plate was calculated as 0.72 or − 2.85 dB. Finally, the efficiencies of the folded dipole and patch-shaped metasurface from the measurement results were calculated with 60.00% and 59.87%. To be compared with the measured efficiencies, we also calculated the deflection efficiency using a ratio between the reflected power integrated along a beam at 70° and the total incident power in simulations^31^. Here, the total incident power is the power density of incident wave times the area of the metasurface. The simulation results also showed similar efficiencies of 53.15% and 54.00% for the folded dipole and the patch metasurfaces. Nearly 6% difference between the measurement and simulation mainly can be attributed to power capturing area difference between the aperture antenna and numerical integration, and non-uniformity of wave-front of the antenna in the measurement. The similar efficiencies from both metasurfaces despite lower harmonic reflection levels at − 70° and 0° from the folded dipole metasurface can be explained by high self-confined fields and related ohmic loss in the folded dipole metasurface. The simulations show that the folded dipole structure dissipates 5.06% of the incident power, while the patch-based metasurface loses 1.14% as ohmic loss. Therefore, we can conclude that the balance between the ohmic loss and harmonic levels leads to similar anomalous reflection efficiencies from both metasurfaces.

**Figure 9:**
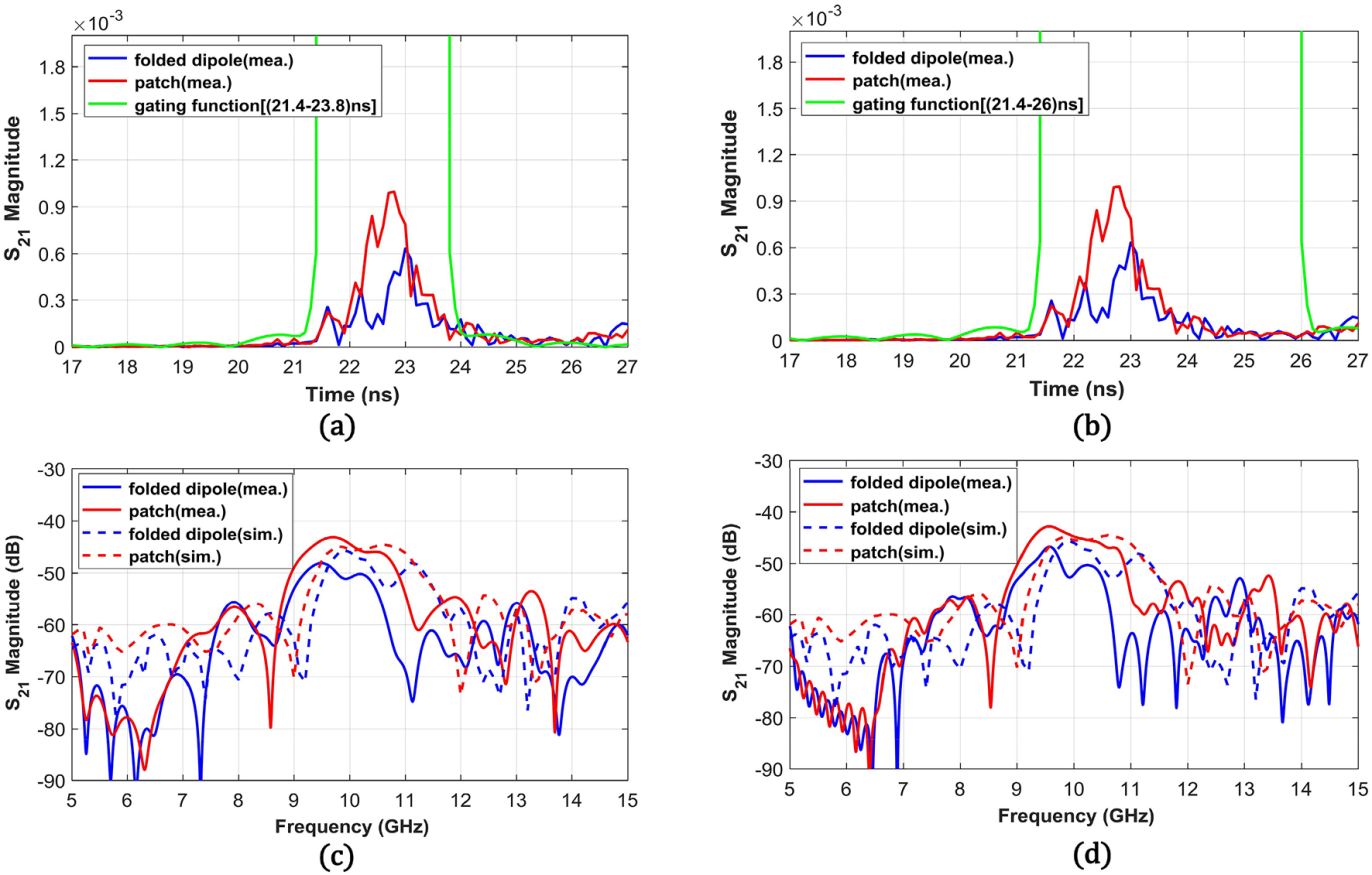
Transmission coefficients (S_21_) at θ = − 70° in (**a**) the time-domain signal with a 2.4 ns-wide gating window (21.4–23.8 ns) for considering only the main peak of reflection from the metasurfaces and (**b**) the time-domain signal with a 4.6 ns-wide gating window (21.4–26 ns) for considering the main peak along with following reflection peaks. Simulation results without the gating and measurement results in the frequency domain converted from the gated time-domain signals using (**c**) the 2.4 ns and (**d**) the 4.6 ns gating windows.

To check the reflection levels at the undesired harmonics, we also analyzed the measurement result at θ = − 70° (n = − 1) and θ = 0° (n = 0) for both metasurfaces. Figure 9a,b shows the time-domain signals measured at θ = − 70° with 2.4 ns (21.4–23.8 ns) and 4.6 ns (21.4–26 ns) gating functions, respectively. Corresponding frequency responses from the gated time-domain signals using the two gating functions are shown in Fig. 9c,d. The simulation results with the adjusted maximum level according to the measured data are also added. The time-domain signals clearly show higher reflection peaks from the patch-based metasurface and a similar trend is also shown in the frequency response near 10 GHz. Figure 9c,d shows that the inclusion of the following reflection peaks using the 4.6 ns-wide gating function increased the reflection level of the patch-based metasurface near 10 GHz but decreased the level of the folded dipole metasurface. The time-domain analysis with different time gating functions demonstrates that the in-band reflection performance from the folded dipole metasurface is maintained even with the ringing effect which is similar to the θ = 70° case.

From Fig. 9d, both metasurfaces show well-matched simulation and measurement S_21_ trends with ~ 0.4 GHz frequency downshift similar to S_21_ signals at θ = 70° case. Overall, the fabricated patch metasurface reflects more than the folded dipole metasurface around 9.6 GHz. Then, we selected the peak values at 9.6 GHz from both metasurfaces for correlation with the simulation values at 10 GHz and found that 4 dB difference from the measurement is higher than 1.13 dB from the simulation. The higher reflection from the patch-based metasurface can also be seen in the time-domain signals in Fig. 9b indicating most of the reflected signals at θ = − 70° arrives after direct reflection at 22 ns. This signal trend means that the reflection to θ = − 70° mainly comes from scattering from the guided waves and scattering at the edges. Therefore, the higher measured harmonic level from the patch metasurface can be attributed to stronger guided waves and edge scattering from fabrication uncertainties e.g., parasitic effect and dielectric constant variation in the substrate. On the other hand, the fabricated folded dipole metasurface provides more correlated reflection levels with the simulation results due to the different reflection mechanism using current excitements inside the antenna structures instead of the guided waves.

**Figure 10:**
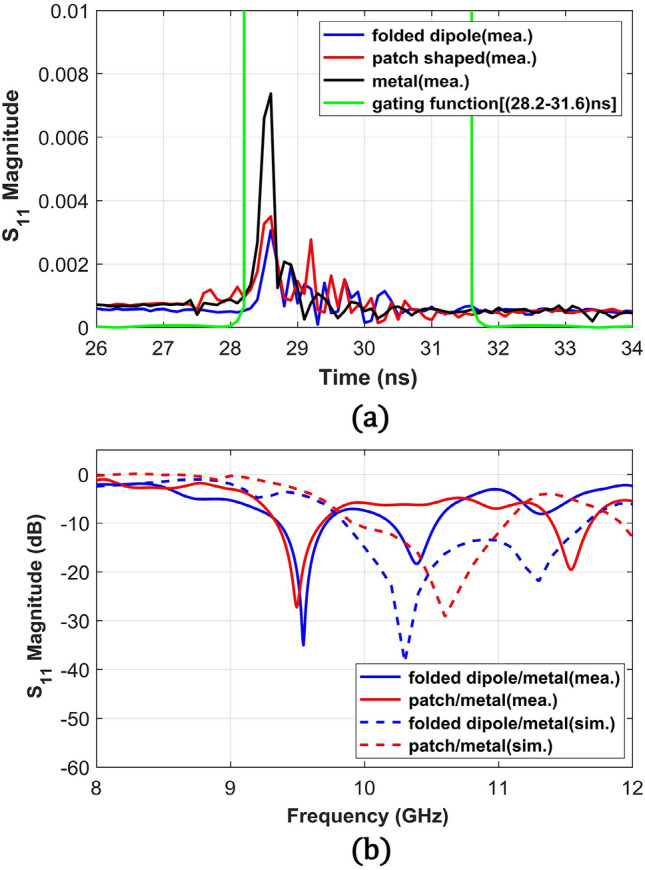
Reflection coefficient (S_11_) magnitude at θ = 0° in (**a**) the time-domain signals from both metasurfaces and the metallic plate with a size of the patch metasurface with a 3.4 ns-wide gating window (28.2–31.6 ns) (**b**) S_11_ magnitude of both metasurfaces normalized with S_11_ from the metallic plate mounted in the normal direction at θ = 0°. Measurement results in the frequency domain were converted from the gated time-domain signals using the 3.4 ns gating window.

Last, to find the reflected signal levels at θ = 0° (n = 0) from the metasurfaces, we measured S_11_ at a port connected to the transmitting antenna. S_11_ from the metallic plates directed toward θ = 0° was also measured as references to verify the correct positioning of the metasurfaces. Figure 10a shows the transformed time-domain signals of all the measured S_11_ data including a metallic plate with a size of the patch metasurface and a 3.4 ns-wide (28.2–31.6 ns) gating function. Due to a round trip (8 m) between the structures and the transmitting antenna, the main peak is located near 28.6 ns, which is later than the other two cases (θ = 70° and − 70°). Different from θ = 70° case, the main reflection peaks from all the structures occur at the same time which means the reflection to θ = 0° from both metasurfaces is mainly due to direct reflection. Therefore, the folded dipole metasurface will have a lower reflection on the normal direction, because most of the directly reflected power is transmitted to the anomalous direction (θ = 70°). Figure 10b depicts the frequency domain S_11_ data from the gated time-domain signal of both metasurfaces that are normalized by the signal levels from the corresponding metallic plates. The frequency responses effectively show that the harmonic suppression minimum ranges lower than − 20 dB for both metasurfaces occur near 9.6 GHz. This frequency coincides with the frequency range for the maximum reflection to 70°. Along with a frequency range of 9.6–10 GHz, the folded dipole-based metasurface maintains minimum 5 dB lower reflection levels compared to the patch-shaped metasurface and the levels are well correlated with 5 dB reflection difference near 10 GHz from the simulations.

## Discussion and conclusion

In this paper, a metasurface composed of folded dipole unit cells was designed to produce efficient reflections at high angles while reducing unnecessary harmonic reflections at 10 GHz and its performance was experimentally demonstrated. Recent papers assumed that surface waves are needed for the perfect reflection at high angles and demonstrated the concept using an electrically large metasurface^23,32^. However, this surface wave can be scattered at the edges in a finite-sized metasurface for practical purposes; therefore, there should be a solution. Because there is no analytical solution and related impedance boundary conditions for surface wave launching, the optimization process was required. This work provides an alternative way to design a reflective metasurface for high angles using folded dipole-based unit cells which provide a lower undesired scattering level. Inside each unit cell, the independent excitement of the looped current leads to the time delay of each unit cell needed for reflection to the high angles. This proposed metasurface also has a lower burden on the optimization in a dense array required for reflection to high angles because the near-field coupling is lower than the conventional patch-based unit cells. Moreover, for the first time, in the metasurface design, we utilized the time-domain signal analysis on the reflected signals from the metasurfaces to understand the anomalous reflection mechanism and the sources of harmonic waves. Finally, we envision the folded dipole-based metasurface as a practical reflect-array design for the millimeter-wave communication link because the structure can be utilized for low harmonic levels in a compact platform.

## Methods

### Electromagnetics simulation

Commercially available ANSYS-HFSS software was used for full-wave numerical simulations of the proposed unit cells and the arrays for the metasurfaces. Unit cell simulations used PEC and PMC boundaries with two wave ports and the propagation length between the unit cell and the ports were de-embedded for reflection phase calculation. Finite array simulation for the metasurface used radiation boundary condition with incident plane wave propagating to the surface normal of the metasurface. The metasurfaces were designed on Rogers 6006, with dielectric thickness = 2.5 mm, $\upvarepsilon _{{\text{r}}}$ = 6.15, and a loss tangent = 0.0019. The metallic structures were modeled with copper with thickness = 35 μm and $\upsigma$ = 5.8 × 10^7^ S/m.

### Measurement and time-domain analysis

The reflection performance of the metasurfaces was measured with the transmission coefficient (S_21_) using a vector network analyzer (Anritsu MS46122B), which was connected to two standard horn antennas (Flann Microwave 16240-20) with 20 dBi gain at 10 GHz as a transmitter and a receiver. The transmitter sits right in front of the metasurface at 4 m (133.33 λ) far, while the receiver antenna changed angles (70 and − 70°) to the surface normal of the metasurface with 2 m (66.67 λ) distance. An industrial laser alignment system (Easy-Laser 22152) was used to align the metasurfaces and the horn antennas. The reflected power to the reflection angle of 0° from the metasurface was measured with the reflection coefficient (S_11_) from a port connected to the transmitter antenna (4 m distant with the metasurface).

With this setup, the frequency domain s-parameters (5–10 GHz) with 1601 sampling points from the network analyzer were measured. For time-domain analysis, a MATLAB code was developed to conduct IFFT (Inverse Fast Fourier Transform), FFT (Fast Fourier Transform), and Kaiser Window-based gating. In the code, the gating start time and the gating span were controlled as variables and $\upbeta$ for the Kaiser window was maintained as 6 for all the cases.

### Reflection efficiency calculation

The reflection efficiency of the metasurface for the reflection angle of 70° was calculated using a ratio of S_21_ values from the metasurface (S_21,m_) and the metallic plate (S_21,p_). The ratio is divided by a correction factor $(\upvarepsilon _{0} )$ and the equation for the efficiency is

5 $$\varepsilon_{r} = \frac{1}{{\varepsilon_{0} }}\frac{{ \left| {S_{21,m} } \right|}}{{\left| {S_{21,p} } \right|}}$$

S_21,m_ and S_21,p_ were measured at the same transmitter (θ = 0°) and the receiver antenna (θ = 70°). The metasurface faced the transmission antenna, but the metallic plate was tilted with 35° along the θ direction. Due to the tilted position of the metallic plate, its effective area became larger compared to the metasurface; thus, a correction factor $(\varepsilon_{0} )$ should be applied. The equations for the correction factor $(\varepsilon_{0} )$ are^23,24^

6 $$E_{scm} = \hat{x}\frac{{jk_{0} E_{0} }}{{2\pi \sqrt {cos\theta } }}\int_{{ - a/2^{cos\theta } }}^{{ - a/2^{cos\theta } }} {\int_{ - b/2}^{ - b/2} {\frac{{e^{{ - jk_{0} r_{1} \left( {u,x} \right)}} }}{{r_{1} \left( {u,x} \right)}}dudx} }$$

where $$r_{1} \left( {u,x} \right) = \sqrt {u^{2} + x^{2} + R^{2} }$$

7 $$E_{scp} = - \hat{x}\frac{{jk_{0} E_{0} cos\upphi }}{2\pi }\int_{ - a/2}^{a/2} {\int_{ - b/2}^{b/2} {\frac{{e^{{jk_{0} \left[ {usin\upphi - r_{2} \left( {u,x} \right)} \right]}} }}{{r_{2} \left( {u,x} \right)}}dudx} }$$

where $$r_{2} \left( {u,x} \right) = \sqrt {u^{2} + x^{2} + 2uRsin\upphi + R^{2} }$$

8 $$\varepsilon_{0} = \left| {\frac{{E_{scm} }}{{E_{scp} }}} \right|$$

Here, the correction factor $(\varepsilon_{0} )$ is the absolute value ratio between scattered electric fields from the metasurface $(|E_{scm} |)$ and the metallic plate $(|{{E}_{\text{scp}}}|)$. The approximations of the surface current to achieve the electric fields in Eqs. (6) and (7) are from^23^. In Eqs. (6) and (7), ${\text{E}}_{0}$ is the electric field of the incident wave, k_0_ is the wavenumber in free space, $a$ and $b$ are the dimensions of the reflective surfaces ($a$ = 220.5 mm and $b$ = 108 mm or 127 mm), and $\upphi$ is 35°, the tilted angle of the metallic plate. $r_{1,2} \left( {u,x} \right)$ are the distances from the receiving antenna to the surface of the metasurface and the metallic plate, respectively. $u$ represents a position along a 35°-tilted axis with respect to the y-axis, as shown in Fig. 4d and R is the distance between the center of metasurface and the receiving horn antenna (2 m).
